# Combination Effects of Antimicrobial Peptides

**DOI:** 10.1128/AAC.02434-15

**Published:** 2016-02-26

**Authors:** Guozhi Yu, Desiree Y. Baeder, Roland R. Regoes, Jens Rolff

**Affiliations:** a Evolutionary Biology, Institut für Biologie, Freie Universität Berlin, Berlin, Germany; b Institute of Integrative Biology, ETH Zurich, Zurich, Switzerland; c Berlin-Brandenburg Institute of Advanced Biodiversity Research (BBIB), Berlin, Germany

## Abstract

Antimicrobial peptides (AMPs) are ancient and conserved across the tree of life. Their efficacy over evolutionary time has been largely attributed to their mechanisms of killing. Yet, the understanding of their pharmacodynamics both *in vivo* and *in vitro* is very limited. This is, however, crucial for applications of AMPs as drugs and also informs the understanding of the action of AMPs in natural immune systems. Here, we selected six different AMPs from different organisms to test their individual and combined effects *in vitro*. We analyzed their pharmacodynamics based on the Hill function and evaluated the interaction of combinations of two and three AMPs. Interactions of AMPs in our study were mostly synergistic, and three-AMP combinations displayed stronger synergism than two-AMP combinations. This suggests synergism to be a common phenomenon in AMP interaction. Additionally, AMPs displayed a sharp increase in killing within a narrow dose range, contrasting with those of antibiotics. We suggest that our results could lead a way toward better evaluation of AMP application in practice and shed some light on the evolutionary consequences of antimicrobial peptide interactions within the immune system of organisms.

## INTRODUCTION

Combinations of drugs can result in three different forms of interactions: synergism, additivity, and antagonism (1–4); i.e., the effect of two drugs combined is stronger, equal, and weaker than that of the individual drug in the equivalent dose, respectively. Combination treatment is supposed to potentially eliminate resistant strains, delay the evolution of drug resistance, reduce the dosage of individual drugs, and hence, diminish side effects (3, 5, 6). A few recent studies, however, report that the success of combination therapy is context dependent, particularly when targeting both sensitive and resistant strains with a combination of drugs of unknown interaction (7–9). These results demonstrated that synergistic drug pairs can efficiently eradicate bacteria but exacerbate selection of resistance, while antagonistic drug pairs showed the reverse trends.

Various methods have been developed to address the efficacy of mostly two-way drug combinations (1, 2). One of the most commonly used approaches in both theoretical and applied research is Loewe additivity (2, 9–11). Here, the effect of two drugs in combination is determined by the sum of ratios of concentrations of drugs in combination divided by concentration of drugs used individually. Note that both the individual drug concentrations and the combined concentrations have the same effect on bacterial growth; we call these concentrations isoeffective concentrations. Theoretically, if the isoeffective concentrations of equivalent effect level achieved in a matrix of gradients of concentrations were connected by line, a concave line represents synergism, while a convex line represents antagonism (2, 12, 13). Recently, a mechanism-free approach was used successfully to predict the outcome of three antibiotics on the interaction between all three possible two-way combinations (14, 15), but the results do not particularly address the question about the nature of interaction (synergism, additivity, or antagonism). How these approaches can be used for a new class of antimicrobials, antimicrobial peptides (AMPs), is basically unknown. Studies on combinatorial effects of antimicrobial peptides, especially within a pharmacodynamics framework, are scarce (16, 17).

Antimicrobial peptides (AMPs), which form an important component of immune defenses in multicellular organisms (18, 19), have been proposed and are being used as new antibiotic drugs. Some AMPs are already commercially available and ready to be applied in clinical practice to replace or accompany conventional antibiotics (20). Additionally, they are supposed to be less likely to induce resistance and mutagenesis in the natural environment, although resistant strains can be obtained under intensive selection in the laboratory (21–23). When AMPs are used in medical applications, they necessarily interact with the patient's own AMPs. Some experimental studies have addressed the effect of individual pairs of AMPs within the context of innate immunity. Coexpressed AMPs on frog skin, PGLa and magainin-2, are synergistic when applied to both Escherichia coli and tumor cells (16). Moreover, AMPs from mammals (17) and insects (24, 25) were shown to synergize. Hence, understanding general principles of AMP interaction will also contribute to our understanding of interactions of AMPs as immune effectors.

**FIG 1:**
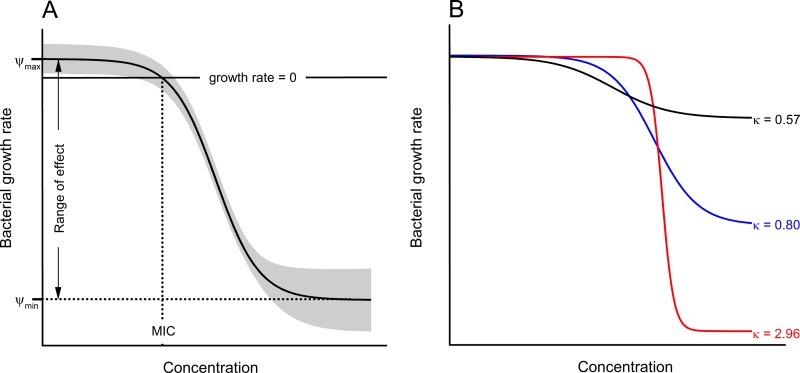
Schematic illustration of four parameters, MIC, ψ_max_, ψ_min_, and κ, predicted by the Hill function. The MIC is estimated by the lowest concentration that inhibits the growth of the whole treated bacterium population. ψ_max_ and ψ_min_ represent the maximal and minimal growth rates of bacteria under gradients of drug treatment, respectively. κ predicts the shape and slope of the pharmacodynamic curve; the higher the κ value, the steeper the pharmacodynamic curve.

Here, we take a pharmacodynamic approach to study the combination effects of AMPs and with combinations of two and three AMPs. Pharmacodynamics capture the functional relationship between drug dosage and bacterial growth or death. We use a modeling approach based on the Hill function (26–28). This model estimates four parameters: MIC, κ, ψ_min_, and ψ_max_ (Fig. 1A). The minimal concentration at which antibiotic substances can inhibit growth of bacteria is MIC; κ depicts the steepness of the curve relating bacterial growth to drug concentration (Fig. 1B); ψ_min_ and ψ_max_ represent the minimum and maximum growth rates of bacteria, respectively. We studied the pairwise and three-way interactions of AMPs using this pharmacodynamic approach embedded in a Loewe additivity framework. According to the work of Loewe, this was achieved by using either one-half (pairwise) or one-third (three-way) of the concentration of each individual drug (see Materials and Methods) (10). We examined the nature of the interactions, synergism or antagonism, and the concentration dependency of the killing. Using a derivative of the human AMP LL-37 enabled us to study interactions between AMPs expressed by patients' innate immunity and AMPs employed as drugs.

## MATERIALS AND METHODS

### Bacteria and media.

Escherichia coli MG1655 was grown in Luria-Bertani (LB) broth at 37°C with aeration at 220 rpm in 50-ml tubes. Two hundred microliters of overnight culture was resuspended into 15 ml fresh LB broth, cultured under the same conditions for an additional 2 h, and then used for subsequent assays. Mueller-Hinton (MH) broth was used for the assay of MICs and time-killing curves.

### AMPs and antibiotics.

We used six different AMPs from different classes of organisms that are commercially available (AnaSpec): cecropin A (Cec) (insect), LL 19-27 (LL) (mammal), melittin (Mel) (insect), pexiganan (Pex) (synthesized AMP, an analog of magainin II; a kind gift of Michael Zasloff), indolicidin (Ind) (mammal), and apidaecin (Api) (insect) (see Table S1 in the supplemental material). These AMPs are effective on either Gram-positive or Gram-negative bacteria (see reviews in references 29 and 30). However, some of these AMPs, e.g., melittin and pexiganan, have anticancer effects (16, 31, 32), which means that they are potentially toxic to human cells, such as erythrocytes. Recently, some low-cell-toxic and serum-stable AMPs have also been under development (33, 34). All these AMPs were dissolved in distilled water with an initial concentration of 1 mg/ml, 5 mg/ml, 10 mg/ml, 1 mg/ml, 1 mg/ml, and 25 mg/ml, respectively, as stock solutions. All antibiotics—ampicillin, ciprofloxacin, gentamicin, kanamycin, neomycin, rifabutin, spectinomycin, and tetracycline—were also dissolved in distilled water and made into 10-mg/ml stock solutions. All the solutions of AMPs and antibiotics were stored at −20°C in a dark environment.

### MIC determination.

According to a standard protocol (35), stock solutions of AMPs were diluted in MH broth and then diluted in 96-well plates with a 2-fold gradient, that is, from 0.25 μg/ml to 128 μg/ml. All the gradients of antibiotics were from 0.02 μg/ml to 50 μg/ml, except that the gradient of ciprofloxacin was from 0.002 μg/ml to 1 μg/ml. Approximately 5 × 10^5^ log-phase bacteria were added to each well. A positive control containing MH broth and bacteria and a negative control containing only MH broth were included in each plate, and plates were incubated at 37°C overnight.

### Measuring killing curves.

To estimate killing curves of each AMP and all possible combinations of AMPs, 100× MICs of AMPs were combined as a volume ratio of 1:1 and 1:1:1 in two-AMP combinations and three-AMP combinations; hence, the concentrations of individual drugs are halved or reduced by two-thirds. Thus, Loewe additivity would result in a MIC of any one combination equal to the MIC of the individual drugs (see equations 5 and 6). Thus, 21 two-AMP combinations and 20 three-AMP combinations were generated. An AMP(s) was diluted, starting with 100× MIC, in a 96-well plate to form a 2-fold gradient of concentrations, and 2 × 10^6^ log-phase bacteria were added to a total volume of 100 μl. The plates were incubated at 37°C. Killing was assessed within 1 h, as killing by AMPs is very fast (36, 37). Ten microliters of a mixture of AMPs and bacteria was taken out every 20 min and then immediately diluted in saline solution and plated on the solid agar plates. These solid agar plates were transferred into a 37°C incubator and cultured overnight for CFU determination. The limit of detection in our system is 100 CFUs.

### Modeling killing curves.

To model the killing curve, the relationship between the concentration of AMP(s) and the killing and/or growth rate of exposed bacteria, we used a Hill function (26):

(1) $$\mu (a)=E_{\max\;}\frac{{\left(a/{\text{EC}}_{50}\right)}^{\kappa }}{1+{\left(a/{\text{EC}}_{50}\right)}^{\kappa }}$$

Here, μ(*a*) is the killing rate at a given concentration of AMP(s); *a* is a given concentration; *E*_max_ is the maximal killing rate of the given AMP(s). κ is the Hill coefficient. We then defined growth rate ψ(*a*) as follows:

(2) $$\psi (a)=\psi_{\max\;}-\mu (a)$$

Here, ψ_max_ is the maximal growth rate of bacteria without AMP(s). The maximum effect of AMP(s) is defined by

(3) $$E_{\max\;}=\psi_{\max\;}-\psi_{\min\;}$$

Thus, the effect of AMP(s) in a given concentration, μ(*a*), can be rewritten as

(4) $$\mu (a)=\frac{\left(\psi_{\max\;}-\psi_{\min\;}\right){\left(a/\text{zMIC}\right)}^{\kappa }}{{\left(a/\text{zMIC}\right)}^{\kappa }-\psi_{\min\;}/\psi_{\max\;}}$$

zMIC is the estimated MIC. Growth rate and killing rate of bacteria are estimated from the time-kill curves as the change of CFU over time by using generalized linear regression. The data for CFU were all log transformed. The start point of linear regression was the first measurement. We then fitted the growth rate and killing rate with equation 4 based on the Markov chain Monte Carlo (MCMC) method using *rjags* (38) in R (39) and generated the pharmacodynamic curves.

### Determining the effect of combination.

Based on the Hill function and isobologram analysis, we obtained the isoeffective concentrations of single drugs and of combinations which achieved a given percentage of their maximal effects or fraction level. The Loewe additivity model defines the additive effect of isoeffective combinations of drugs that result in a certain effect. For example, the combination of drug A and drug B in the isoeffective concentrations, which are *C*_isoA_ and *C*_isoB_, can achieve a level of effect which can also be achieved individually by drug A or drug B with a concentration of *C*_A_ or *C*_B_, respectively. Mathematically, the combination effect of drug A and drug B is defined as follows:

(5) $$\text{CI}=\frac{C_{\text{isoA}}}{C_{\text{A}}}+\frac{C_{\text{isoB}}}{C_{\text{B}}}$$

For three-drug combinations

(6) $$\text{CI}=\frac{C_{\text{isoA}}}{C_{\text{A}}}+\frac{C_{\text{isoB}}}{C_{\text{B}}}+\frac{C_{\text{isoC}}}{C_{\text{C}}}$$

Additive combination effects were then defined by a combination index (CI) equal to 1, antagonism was defined as a CI greater than 1, and synergism was defined as a CI lower than 1.

## RESULTS

### Killing and pharmacodynamic curves.

We tested the *in vitro* effects of single AMPs, two-AMP combinations, and three-AMP combinations on E. coli. All killing curves were obtained by counting viable CFU after treatment (see Fig. S1 in the supplemental material). In most cases, the number of surviving bacteria drastically decreased as a function of time at higher concentrations while slightly increasing at lower concentrations. Killing occurred very quickly at higher concentrations in our system (i.e., bacterial densities below the limit of detection).

**FIG 2:**
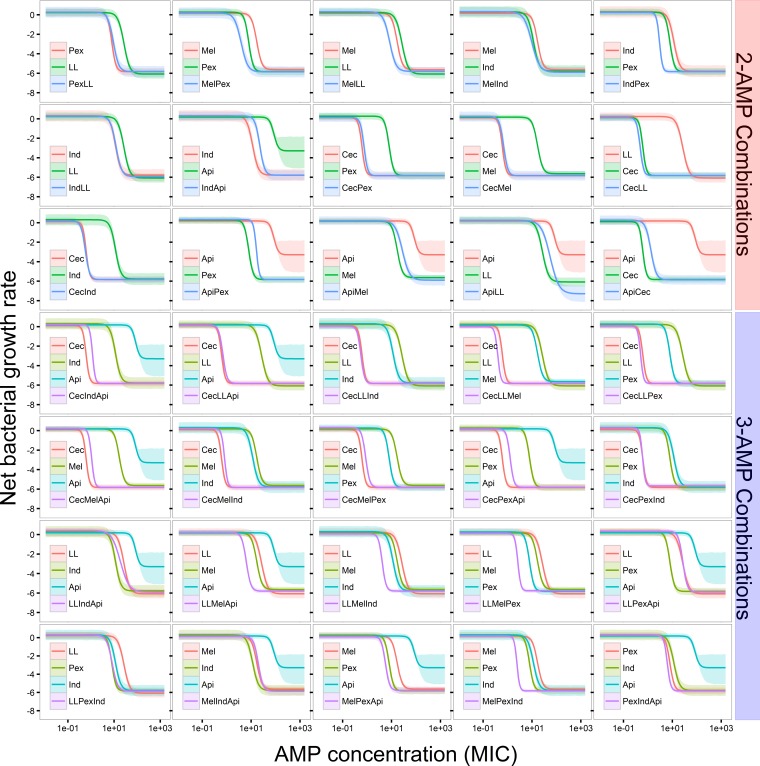
Pharmacodynamic curves of AMPs. The pharmacodynamic curves of AMPs were obtained by fitting killing curves to the Hill function (see equation 4). Combinations of two or three AMPs were differentiated. The curves illustrate the effects (reflected as net bacterial growth rate) of increasing the concentrations of AMP(s). The ribbon represents the 95% confidence interval.

**FIG 3:**
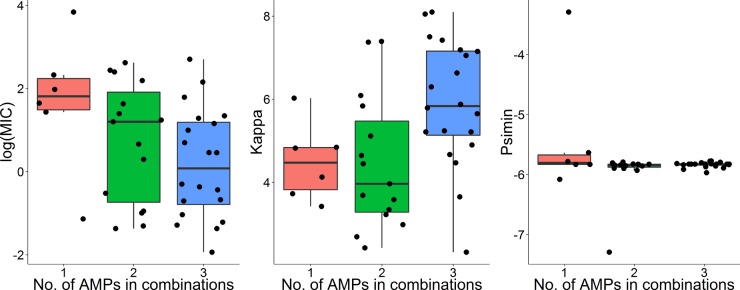
Variations of MIC, κ, and ψ_min_ in the Hill function predicted by the MCMC method. Results showed that these parameters vary among combinations with different numbers of AMPs. MICs declined with increasing numbers of AMPs in combination (ANOVA, *F*_1,39_ = 6.647, *P* = 0.0138); combinations with three AMPs had the highest κ values (ANOVA, *F*_1,39_ = 7.447, *P* = 0.00935). ψ_min_ (Psimin) did not show significant differences among single AMPs and two- and three-AMP combinations (ANOVA, *F*_1,39_ = 1.855, *P* = 0.181).

The four pharmacodynamic parameters, MIC, κ, ψ_max_, and ψ_min_, were estimated by the MCMC method using the generalized linear regression fitted killing rate as a function of concentrations of AMP(s) (Fig. 2 and 3; also see Table S2 in the supplemental material). Notably, all the single AMPs and two- and three-AMP combinations had almost the same ψ_min_ (analysis of variance [ANOVA], ψ_min_, *F*_1,39_ = 1.855, *P* = 0.181) (Fig. 3; see also Table S2 in the supplemental material). ψ_max_ values were also identical in different treatments as the growth rate of bacteria in low concentrations of AMP(s) was presumably close to the natural growth rate. Two pharmacodynamic parameters, MIC and κ, varied among different treatments, with three-AMP combinations having the lowest MICs and the highest κ values (ANOVA, MIC, *F*_1,39_ = 6.647, *P* = 0.0138; κ, *F*_1,39_ = 7.447, *P* = 0.00935) (Fig. 3; see also Table S2 in the supplemental material). All the treatments showed nearly the same pharmacodynamic trend: a sharp decrease of net bacterial growth with an increasing concentration of AMP(s) as depicted by κ.

### Most AMP combinations are synergistic, but synergy is stronger in three-AMP combinations.

**FIG 4:**
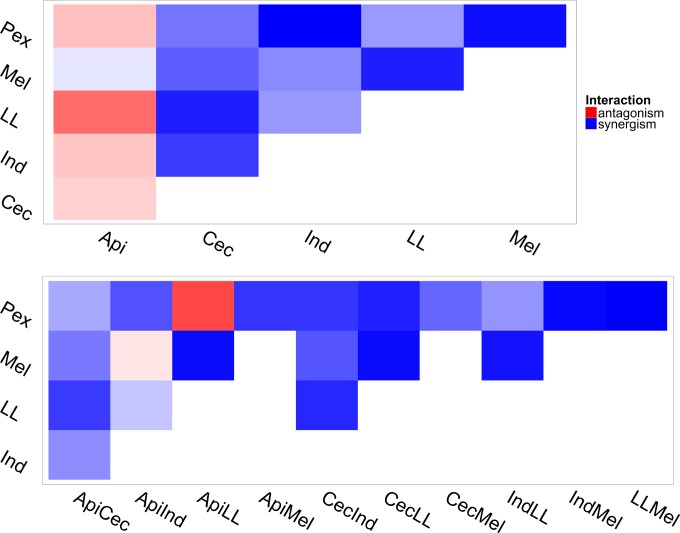
Combination index of AMPs applied at concentrations which can achieve 50% of their maximal effect (E_50_). At E_50_, all the combinations with Api (except the combination of Api and Mel) showed antagonistic effects in two-AMP combinations, but only two combinations, ApiIndMel and ApiLLPex, showed antagonistic effects in three-AMP combinations. The gradient of colors represents different levels of each interaction.

To determine the interaction of AMPs, we used Loewe additivity (see equations 5 and 6). The combination index was calculated for concentrations between 5% and 95% of the maximal effect (equation 3). For two-AMP combinations, we found that most of the two-AMP combinations (67%) were completely synergistic (combination indexes were lower than 1) within the effect range, except for the combination of apidaecin and LL 19-27 (ApiLL), which was antagonistic across the whole range; the combinations PexApi and IndApi were antagonistic in low-concentration combinations but synergistic in high-concentration combinations (Fig. 4; also see Fig. S2 in the supplemental material). However, the combinations CecApi and MelApi had a reverse trend, as they were synergistic in lower-concentration combinations and antagonistic in higher-concentration combinations (Fig. 4; see also Fig. S2 in the supplemental material). Eighty-five percent of three-AMP combinations were completely synergistic within the effect range while the combination LLPexApi was completely antagonistic; LLIndApi showed synergistic effects in lower-concentration combinations and antagonistic effects in higher-concentration combinations, but MelIndApi had the reverse trend (Fig. 4; see also Fig. S2 in the supplemental material).

**FIG 5:**
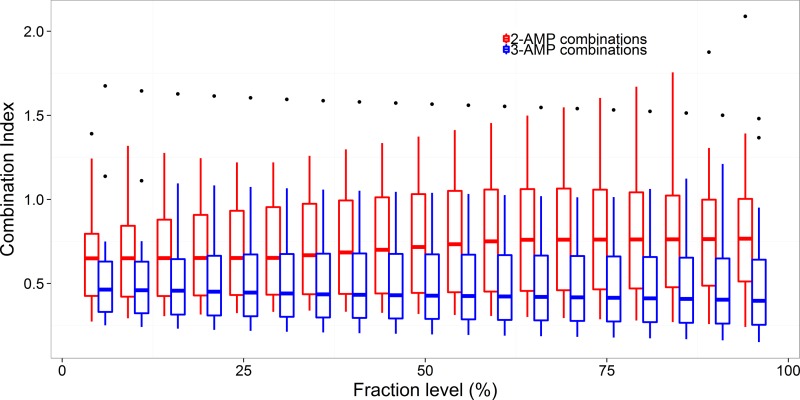
Combination index of fraction level within the effect range. Three-AMP combinations are more significantly synergistic than two-AMP combinations (Student's *t* test, *t* = 8.2016, df = 606.57, *P* = 1.42e−15). The combination index did not vary within different effect ranges in both two-AMP and three-AMP combinations (ANOVA, *F*_1,661_ = 1.332, *P* = 0.2488). Black dots denote outliers.

Another interesting finding is that three-AMP combinations generally have stronger effects than do two-AMP combinations at a given fraction level within the effect range. The average combination indexes of three-AMP combinations were 30% lower than those of two-AMP combinations (Student's *t* test, *t* = 8.2016, df = 606.57, *P* = 1.42e−15) (Fig. 5). We observed no differences between effects of different fractions for the three-way interactions (ANOVA, *F*_1,661_ = 1.332, *P* = 0.2488) (Fig. 5).

### Relationship between κ values and selection.

**FIG 6:**
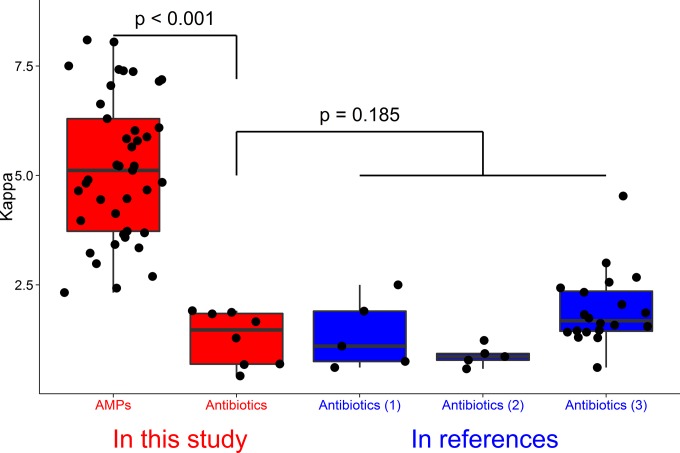
Comparison of κ values of AMPs and antibiotics in our experiment and other, similar experiments. With similar experimental methods, conditions of measurements, and mathematical models, κ values of antibiotics in our experiment are similar to those of antibiotics in other experiments (ANOVA, *F*_1,36_ = 1.591, *P* = 0.215). However, κ values of AMPs are significantly higher than those of antibiotics both in our experiment and in other experiments (ANOVA, *F*_1,77_ = 150.5, *P* < 0.001). Data in boxes “Antibiotics (1),” “Antibiotics (2),” and “Antibiotics (3)” are from references 26, 40, and 11, respectively.

We compared the κ values of different combinations of AMPs and between AMPs and antibiotics. κ values are higher the more AMPs that are combined. We also found that κ values of AMPs are significantly higher (ANOVA, *F*_1,77_ = 150.5, *P* < 0.001) (Fig. 6) than those of antibiotics, for data obtained in both our laboratory and other laboratories (ANOVA, *F*_1,36_ = 1.591, *P* = 0.215) (Fig. 6).

## DISCUSSION

Pharmacodynamic approaches have been frequently applied to conventional antibiotics (2, 11, 26, 40). A good understanding of how antimicrobial peptides eradicate bacteria in complex systems not only relies on the molecular mechanisms of killing but, importantly, necessitates investigation of pharmacodynamics *in vitro*, as done here and *in vivo* (C. Zanchi, P. R. Johnston, and J. Rolff, unpublished data). Generally, the maximal killing values were almost identical in treatments with all the AMPs and their two- and three-way combinations, which means that high concentrations of an AMP(s) may eradicate bacteria with similar efficiencies. Due to fast killing of AMPs and the limit of detection in our system, the real maximal killing rate might be masked at higher concentrations, e.g., concentrations in which the limit of detection is reached within 20 min. However, the MIC and κ significantly varied among single AMPs and two- and three-AMP combinations. As numbers of AMPs increased in combination, the MIC of that combination decreased, with the lowest value seen in three-AMP combinations, and κ was much higher in three-AMP combinations (Fig. 3). More AMPs combined with lower MICs demonstrate that the absolute quantity of AMP needs to be decreased to achieve the same killing. Higher κ values in combined AMPs result in a drastic decrease in bacterial killing rate within a narrow range of concentrations of the AMP(s). The combination of AMPs might improve the efficiency of bacterial killing. Taken together, the decreasing MIC and increasing κ values in combinations with increasing numbers of AMPs suggest that synergism is common in AMP combinations (17, 24, 41).

We observed broad synergistic effects in almost all the two- and three-way combinations. Although some AMPs, like apidaecin, had a relatively weak effect with a high MIC, the killing could still be enhanced by adding one or more AMPs with stronger individual effects. Synergism, albeit not within a pharmacodynamics framework, has been reported for 2-way combinations of antibiotics (8, 42), AMPs (16, 17, 24), antimicrobial peptoids (43), antibiotics and AMPs (44, 45), and AMPs and antimicrobial peptoids (43). The AMPs, originating from different species in our experiment, showed robust synergism, which suggests a general effect.

The molecular mechanisms of interaction, especially antagonisms, of AMPs are largely unknown. As most AMPs target the membrane of pathogens, their interactions are unlikely to directly disrupt the metabolic network in the cell like certain antibiotics. A recent study suggested that synergism was caused by the conjugation of coapplied AMPs, which form a supermolecule and better-stabilized pores (41). This is also confirmed by chemically conjoined synthesized peptides (46). Furthermore, pore-forming peptides can also assist other coapplied transmembrane AMPs to quickly invade bacterial cells and substantially interrupt the metabolism (47).

In our pharmacodynamic model, the important parameter κ depicts the steepness of the pharmacodynamic curve and is a measure of the sensitivity of the response of the bacteria to changes in the concentrations of the antimicrobial substances. A steeper pharmacodynamic curve with higher κ values illustrates that bacteria are very sensitive to the change of concentrations of AMPs and antibiotics, which means that the given antibiotic substance (e.g., combinations of AMPs) has a narrower range of concentrations exerting selection on bacteria.

Additionally, κ value could be an important indicator of resistance selection of given antibiotic agents. Traditionally, the presence of antimicrobial substances above the MIC is thought to favor resistant strains. The mutant selection window (MSW) is defined as the difference in the MICs of a resistant and a susceptible strain (48, 49). Thus, the MSW can be specifically defined as the range between the concentration killing all the sensitive strains and the concentration killing all the resistant strains. Additionally, MSW also can be defined as a range of concentrations which can *de novo* select mutant strains from a completely sensitive population (50–52). Higher κ values in combinations of AMPs denote a steeper pharmacodynamic curve, which means that the range of concentrations selecting resistance—the MSW—can be narrowed. Especially, the sub-MIC part of the MSW is predicted to be very small for high κ values. A previous theoretical study also demonstrated that the synergistic contribution of the immune system can potentially narrow the mutant selection window of antibiotics (53). We observed a synergistic interaction in combinations of AMPs that mirrors, in the case of LL 19-27, interactions between the immune system and drugs. Higher κ values of AMPs than of antibiotics might partially explain the fact that bacteria are unlikely to develop resistance to AMPs in nature, although resistant strains can emerge under intensive selection in the laboratory (21, 54).

### Conclusion.

Our study suggests that the synergistic effect between AMPs may be a common phenomenon, as we observed strong synergistic interactions in two-AMP and three-AMP combinations. Interestingly, these three-AMP combinations are even more synergistic than two-AMP combinations. If synergistic interactions of AMPs are ubiquitous, than two practical implications arise: (i) AMPs that strongly synergize with host AMPs should be utilized and (ii) combinations provide the opportunity to reduce side effects, as they lead to an overall reduction in dosage. In the context of innate immunity, selection should favor organisms producing AMP cocktails. This can be considered a cost-efficient way of reducing bacterial loads in a host (19, 55).

Long-lasting coexpression of combinations of AMPs has been recorded in Xenopus laevis (56) and Tenebrio molitor (19), where it is correlated with metabolic suppression. Thus, evolving a more efficient killing system based on a relatively energy-constrained system, which expresses only a limited number of AMPs, is necessary and practical. A function of synergism among AMPs is one of the possible ways to mitigate the costs.

Our results have some implications for the applied use of AMPs as drugs. The production of AMPs is currently expensive (20). The broad synergism observed in our experiment means that combined applications of AMPs could also reduce the consumption of total AMPs just as in the immune system, which could eventually save costs of treatment and reduce toxicity. As humans express AMPs such as LL-37 in their innate immune system, synergisms between these AMPs and AMPs applied as drugs should be taken into account. In our study, the human AMP derivative LL 17-29 synergized with almost all combinations of AMPs. Though resistance to single AMPs evolves readily *in vitro*, it is might be less likely under combinations (54). It is possible that in some situations combinations delay the development of resistance in medical practice, as pathogens could pay a higher cost to evolve resistance to multidrug treatment (57–60).

## Supplementary Material

Supplemental material

## References

[1] Greco WR, Bravo G, Parsons JC 1995 The search for synergy: a critical review from a response surface perspective. Pharmacol Rev 47:331–385. 7568331

[2] Chou TC 2006 Theoretical basis, experimental design, and computerized simulation of synergism and antagonism in drug combination studies. Pharmacol Rev 58:621–681. doi:10.1124/pr.58.3.10. 16968952

[3] Imamovic L, Sommer MO 2013 Use of collateral sensitivity networks to design drug cycling protocols that avoid resistance development. Sci Transl Med 5:204ra132. doi:10.1126/scitranslmed.3006609. 24068739

[4] Cokol M, Chua HN, Tasan M, Mutlu B, Weinstein ZB, Suzuki Y, Nergiz ME, Costanzo M, Baryshnikova A, Giaever G, Nislow C, Myers CL, Andrews BJ, Boone C, Roth FP 2011 Systematic exploration of synergistic drug pairs. Mol Syst Biol 7:544. doi:10.1038/msb.2011.71. 22068327 PMC3261710

[5] Tamma PD, Cosgrove SE, Maragakis LL 2012 Combination therapy for treatment of infections with gram-negative bacteria. Clin Microbiol Rev 25:450–470. doi:10.1128/CMR.05041-11. 22763634 PMC3416487

[6] Worthington RJ, Melander C 2013 Combination approaches to combat multidrug-resistant bacteria. Trends Biotechnol 31:177–184. doi:10.1016/j.tibtech.2012.12.006. 23333434 PMC3594660

[7] Pena-Miller R, Laehnemann D, Jansen G, Fuentes-Hernandez A, Rosenstiel P, Schulenburg H, Beardmore R 2013 When the most potent combination of antibiotics selects for the greatest bacterial load: the smile-frown transition. PLoS Biol 11:e1001540. doi:10.1371/journal.pbio.1001540. 23630452 PMC3635860

[8] Yeh PJ, Hegreness MJ, Aiden AP, Kishony R 2009 Drug interactions and the evolution of antibiotic resistance. Nat Rev Microbiol 7:460–466. doi:10.1038/nrmicro2133. 19444248 PMC2855488

[9] Chait R, Craney A, Kishony R 2007 Antibiotic interactions that select against resistance. Nature 446:668–671. doi:10.1038/nature05685. 17410176

[10] Loewe S 1953 The problem of synergism and antagonism of combined drugs. Arzneimittelforschung 3:285–290. 13081480

[11] Ankomah P, Johnson PJ, Levin BR 2013 The pharmaco-, population and evolutionary dynamics of multi-drug therapy: experiments with S. aureus and E. coli and computer simulations. PLoS Pathog 9:e1003300. doi:10.1371/journal.ppat.1003300. 23593006 PMC3617031

[12] Ocampo PS, Lazar V, Papp B, Arnoldini M, Abel zur Wiesch P, Busa-Fekete R, Fekete G, Pal C, Ackermann M, Bonhoeffer S 2014 Antagonism between bacteriostatic and bactericidal antibiotics is prevalent. Antimicrob Agents Chemother 58:4573–4582. doi:10.1128/AAC.02463-14. 24867991 PMC4135978

[13] Michel JB, Yeh PJ, Chait R, Moellering RC Jr, Kishony R 2008 Drug interactions modulate the potential for evolution of resistance. Proc Natl Acad Sci U S A 105:14918–14923. doi:10.1073/pnas.0800944105. 18815368 PMC2567468

[14] Wood K, Nishida S, Sontag ED, Cluzel P 2012 Mechanism-independent method for predicting response to multidrug combinations in bacteria. Proc Natl Acad Sci U S A 109:12254–12259. doi:10.1073/pnas.1201281109. 22773816 PMC3409729

[15] Rothschild D, Dekel E, Hausser J, Bren A, Aidelberg G, Szekely P, Alon U 2014 Linear superposition and prediction of bacterial promoter activity dynamics in complex conditions. PLoS Comput Biol 10:e1003602. doi:10.1371/journal.pcbi.1003602. 24809350 PMC4014397

[16] Westerhoff HV, Zasloff M, Rosner JL, Hendler RW, De Waal A, Vaz Gomes A, Jongsma PM, Riethorst A, Juretic D 1995 Functional synergism of the magainins PGLa and magainin-2 in Escherichia coli, tumor cells and liposomes. Eur J Biochem 228:257–264. doi:10.1111/j.1432-1033.1995.00257.x. 7705337

[17] Yan H, Hancock RE 2001 Synergistic interactions between mammalian antimicrobial defense peptides. Antimicrob Agents Chemother 45:1558–1560. doi:10.1128/AAC.45.5.1558-1560.2001. 11302828 PMC90506

[18] Johnston PR, Rolff J 2013 Immune- and wound-dependent differential gene expression in an ancient insect. Dev Comp Immunol 40:320–324. doi:10.1016/j.dci.2013.01.012. 23395998

[19] Johnston PR, Makarova O, Rolff J 2014 Inducible defenses stay up late: temporal patterns of immune gene expression in Tenebrio molitor. G3 (Bethesda, Md.) 4:947–955. doi:10.1534/g3.113.008516. PMC4065263 24318927

[20] Giuliani A, Pirri G, Nicoletto SF 2007 Antimicrobial peptides: an overview of a promising class of therapeutics. Cent Eur J Biol 2:1–33.

[21] Perron GG, Zasloff M, Bell G 2006 Experimental evolution of resistance to an antimicrobial peptide. Proc Biol Sci 273:251–256. doi:10.1098/rspb.2005.3301. 16555795 PMC1560030

[22] Rodriguez-Rojas A, Makarova O, Rolff J 2014 Antimicrobials, stress and mutagenesis. PLoS Pathog 10:e1004445. doi:10.1371/journal.ppat.1004445. 25299705 PMC4192597

[23] Dobson AJ, Purves J, Rolff J 2014 Increased survival of experimentally evolved antimicrobial peptide-resistant Staphylococcus aureus in an animal host. Evol Appl 7:905–912. doi:10.1111/eva.12184. 25469169 PMC4211720

[24] Rahnamaeian M, Cytrynska M, Zdybicka-Barabas A, Dobslaff K, Wiesner J, Twyman RM, Zuchner T, Sadd BM, Regoes RR, Schmid-Hempel P, Vilcinskas A 2015 Insect antimicrobial peptides show potentiating functional interactions against Gram-negative bacteria. Proc Biol Sci 282:20150293. doi:10.1098/rspb.2015.0293. 25833860 PMC4426631

[25] Poppel AK, Vogel H, Wiesner J, Vilcinskas A 2015 Antimicrobial peptides expressed in medicinal maggots of the blow fly Lucilia sericata show combinatorial activity against bacteria. Antimicrob Agents Chemother 59:2508–2514. doi:10.1128/AAC.05180-14. 25666157 PMC4394815

[26] Regoes RR, Wiuff C, Zappala RM, Garner KN, Baquero F, Levin BR 2004 Pharmacodynamic functions: a multiparameter approach to the design of antibiotic treatment regimens. Antimicrob Agents Chemother 48:3670–3676. doi:10.1128/AAC.48.10.3670-3676.2004. 15388418 PMC521919

[27] Zhi JG, Nightingale CH, Quintiliani R 1986 A pharmacodynamic model for the activity of antibiotics against microorganisms under nonsaturable conditions. J Pharm Sci 75:1063–1067. doi:10.1002/jps.2600751108. 3102718

[28] Nolting A, Dalla Costa T, Rand KH, Derendorf H 1996 Pharmacokinetic pharmacodynamic modeling of the antibiotic effect of piperacillin in vitro. Pharm Res 13:91–96. doi:10.1023/A:1016085402278. 8668686

[29] Zasloff M 2002 Antimicrobial peptides of multicellular organisms. Nature 415:389–395. doi:10.1038/415389a. 11807545

[30] Gordon YJ, Romanowski EG, McDermott AM 2005 A review of antimicrobial peptides and their therapeutic potential as anti-infective drugs. Curr Eye Res 30:505–515. doi:10.1080/02713680590968637. 16020284 PMC1497874

[31] Do N, Weindl G, Grohmann L, Salwiczek M, Koksch B, Korting HC, Schafer-Korting M 2014 Cationic membrane-active peptides—anticancer and antifungal activity as well as penetration into human skin. Exp Dermatol 23:326–331. doi:10.1111/exd.12384. 24661024

[32] Moore AJ, Devine DA, Bibby MC 1994 Preliminary experimental anticancer activity of cecropins. Peptide Res 7:265–269. 7849420

[33] Nguyen LT, Chau JK, Perry NA, de Boer L, Zaat SA, Vogel HJ 2010 Serum stabilities of short tryptophan- and arginine-rich antimicrobial peptide analogs. PLoS One 5:e12684. doi:10.1371/journal.pone.0012684. 20844765 PMC2937036

[34] Nguyen LT, Chan DI, Boszhard L, Zaat SA, Vogel HJ 2010 Structure-function studies of chemokine-derived carboxy-terminal antimicrobial peptides. Biochim Biophys Acta 1798:1062–1072. doi:10.1016/j.bbamem.2009.11.021. 20004172

[35] Wiegand I, Hilpert K, Hancock RE 2008 Agar and broth dilution methods to determine the minimal inhibitory concentration (MIC) of antimicrobial substances. Nat Protoc 3:163–175. doi:10.1038/nprot.2007.521. 18274517

[36] Sochacki KA, Barns KJ, Bucki R, Weisshaar JC 2011 Real-time attack on single Escherichia coli cells by the human antimicrobial peptide LL-37. Proc Natl Acad Sci U S A 108:E77–E81. doi:10.1073/pnas.1101130108. 21464330 PMC3080975

[37] Rangarajan N, Bakshi S, Weisshaar JC 2013 Localized permeabilization of E. coli membranes by the antimicrobial peptide cecropin A. Biochemistry 52:6584–6594. doi:10.1021/bi400785j. 23988088 PMC3813965

[38] Plummer M 2014 rjags: Bayesian graphical models using MCMC, R package version 3-13. R Foundation for Statistical Computing, Vienna, Austria http://CRAN.R-project.org/package=rjags.

[39] R Core Team. 2014 R: a language and environment for statistical computing. R Foundation for Statistical Computing, Vienna, Austria http://www.R-project.org/.

[40] Ankomah P, Levin BR 2012 Two-drug antimicrobial chemotherapy: a mathematical model and experiments with Mycobacterium marinum. PLoS Pathog 8:e1002487. doi:10.1371/journal.ppat.1002487. 22253599 PMC3257304

[41] Matsuzaki K, Mitani Y, Akada KY, Murase O, Yoneyama S, Zasloff M, Miyajima K 1998 Mechanism of synergism between antimicrobial peptides magainin 2 and PGLa. Biochemistry 37:15144–15153. doi:10.1021/bi9811617. 9790678

[42] Monzon M, Oteiza C, Leiva J, Amorena B 2001 Synergy of different antibiotic combinations in biofilms of Staphylococcus epidermidis. J Antimicrob Chemother 48:793–801. doi:10.1093/jac/48.6.793. 11733463

[43] Chongsiriwatana NP, Wetzler M, Barron AE 2011 Functional synergy between antimicrobial peptoids and peptides against Gram-negative bacteria. Antimicrob Agents Chemother 55:5399–5402. doi:10.1128/AAC.00578-11. 21859945 PMC3195000

[44] Naghmouchi K, Le Lay C, Baah J, Drider D 2012 Antibiotic and antimicrobial peptide combinations: synergistic inhibition of Pseudomonas fluorescens and antibiotic-resistant variants. Res Microbiol 163:101–108. doi:10.1016/j.resmic.2011.11.002. 22172555

[45] Choi H, Lee DG 2012 Synergistic effect of antimicrobial peptide arenicin-1 in combination with antibiotics against pathogenic bacteria. Res Microbiol 163:479–486. doi:10.1016/j.resmic.2012.06.001. 22705395

[46] He J, Eckert R, Pharm T, Simanian MD, Hu C, Yarbrough DK, Qi F, Anderson MH, Shi W 2007 Novel synthetic antimicrobial peptides against Streptococcus mutans. Antimicrob Agents Chemother 51:1351–1358. doi:10.1128/AAC.01270-06. 17296741 PMC1855471

[47] Brogden KA 2005 Antimicrobial peptides: pore formers or metabolic inhibitors in bacteria? Nat Rev Microbiol 3:238–250. doi:10.1038/nrmicro1098. 15703760

[48] Andersson DI, Hughes D 2014 Microbiological effects of sublethal levels of antibiotics. Nat Rev Microbiol 12:465–478. doi:10.1038/nrmicro3270. 24861036

[49] Day T, Huijben S, Read AF 2015 Is selection relevant in the evolutionary emergence of drug resistance? Trends Microbiol 23:126–133. doi:10.1016/j.tim.2015.01.005. 25680587 PMC4494118

[50] Drlica K 2003 The mutant selection window and antimicrobial resistance. J Antimicrob Chemother 52:11–17. doi:10.1093/jac/dkg269. 12805267

[51] Drlica K, Zhao X 2007 Mutant selection window hypothesis updated. Clin Infect Dis 44:681–688. doi:10.1086/511642. 17278059

[52] Berghaus LJ, Giguere S, Guldbech K 2013 Mutant prevention concentration and mutant selection window for 10 antimicrobial agents against Rhodococcus equi. Vet Microbiol 166:670–675. doi:10.1016/j.vetmic.2013.07.006. 23915992

[53] Handel A, Margolis E, Levin BR 2009 Exploring the role of the immune response in preventing antibiotic resistance. J Theor Biol 256:655–662. doi:10.1016/j.jtbi.2008.10.025. 19056402 PMC5814249

[54] Dobson AJ, Purves J, Kamysz W, Rolff J 2013 Comparing selection on S. aureus between antimicrobial peptides and common antibiotics. PLoS One 8:e76521. doi:10.1371/journal.pone.0076521. 24204634 PMC3799789

[55] Haine ER, Moret Y, Siva-Jothy MT, Rolff J 2008 Antimicrobial defense and persistent infection in insects. Science 322:1257–1259. doi:10.1126/science.1165265. 19023083

[56] Bevins CL, Zasloff M 1990 Peptides from frog skin. Annu Rev Biochem 59:395–414. doi:10.1146/annurev.bi.59.070190.002143. 2197979

[57] Andersson DI, Hughes D 2010 Antibiotic resistance and its cost: is it possible to reverse resistance? Nat Rev Microbiol 8:260–271. doi:10.1038/nrmicro2319. 20208551

[58] Andersson DI 2006 The biological cost of mutational antibiotic resistance: any practical conclusions? Curr Opin Microbiol 9:461–465. doi:10.1016/j.mib.2006.07.002. 16890008

[59] Hall AR, Angst DC, Schiessl KT, Ackermann M 2015 Costs of antibiotic resistance—separating trait effects and selective effects. Evol Appl 8:261–272. doi:10.1111/eva.12187. 25861384 PMC4380920

[60] Vogwill T, MacLean RC 2015 The genetic basis of the fitness costs of antimicrobial resistance: a meta-analysis approach. Evol Appl 8:284–295. doi:10.1111/eva.12202. 25861386 PMC4380922

