# Supplementary material for: Combination Effects of Antimicrobial Peptides
Source: Antimicrob Agents Chemother. 2016 Feb 26;60(3):1717–24. doi: 10.1128/AAC.02434-15 (PMC4775937; doi:10.1128/AAC.02434-15)
Supplement: Supplemental material [file supp_60_3_1717__index.html]

Supplemental material 

# The More The Better? Combination Effects of Antimicrobial Peptides

## Supplemental material

- Supplemental file 1 -

  Supplemental Figures S1 and S2 and Tables S1 and S2

  PDF, 942K
