# Supplementary material for: Combination Effects of Antimicrobial Peptides
Source: Antimicrob Agents Chemother. 2016 Feb 26;60(3):1717–24. doi: 10.1128/AAC.02434-15 (PMC4775937; doi:10.1128/AAC.02434-15)
Supplement: Supplemental material [file AAC.02434-15_zac003164937so1.pdf]

Figure S1

The killing curve of *E.coli* under treatment of different AMPs and their combinations in different concentrations. The AMPs and their combinations are marked on each panel. Numbers behind represent different biological replicates.

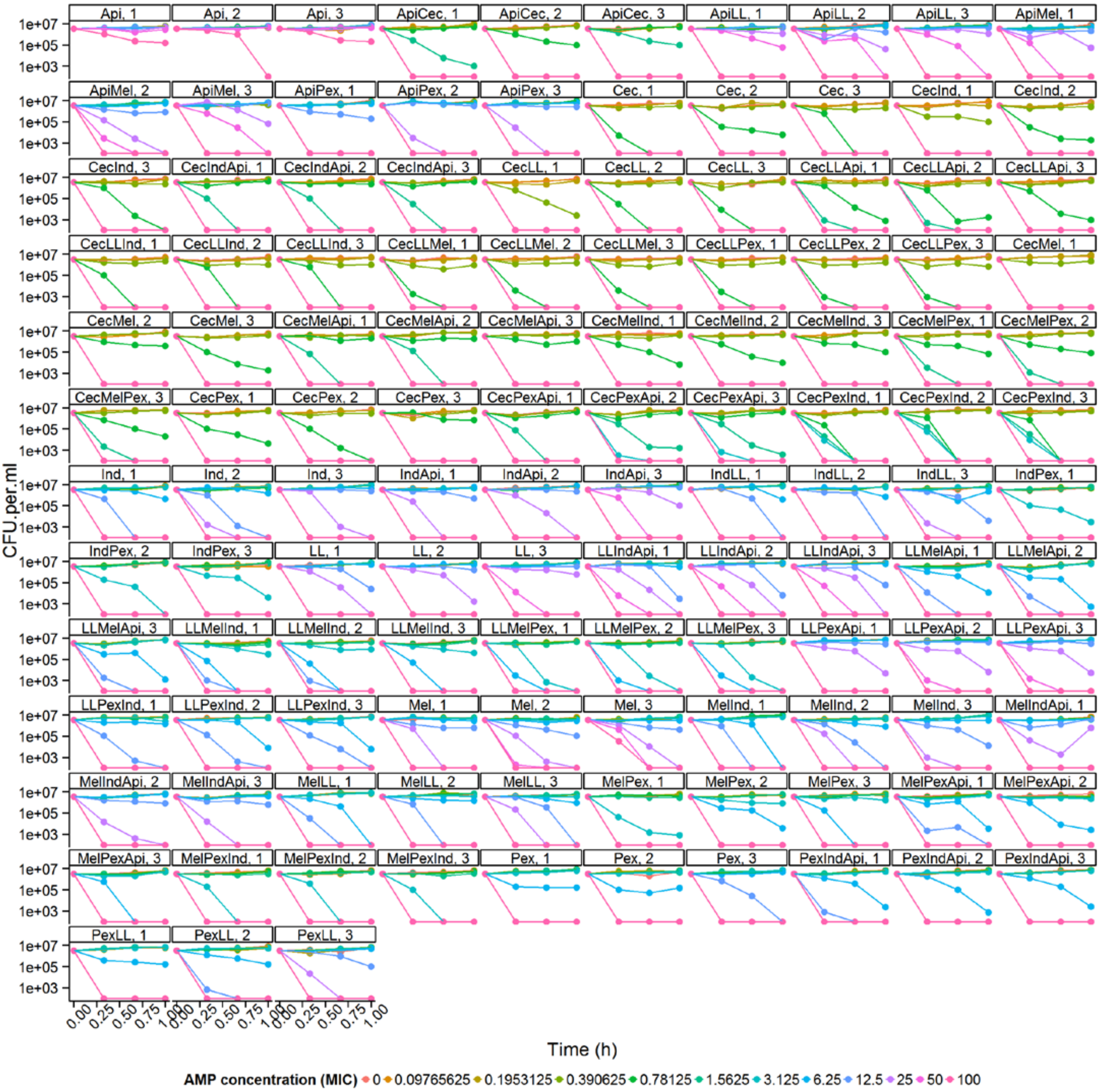

Figure S2

Combination Index of different fraction level within the range of effect (see Figure 1). Values above, below and on one represent synergism, antagonism and additivity in a given combination, respectively.

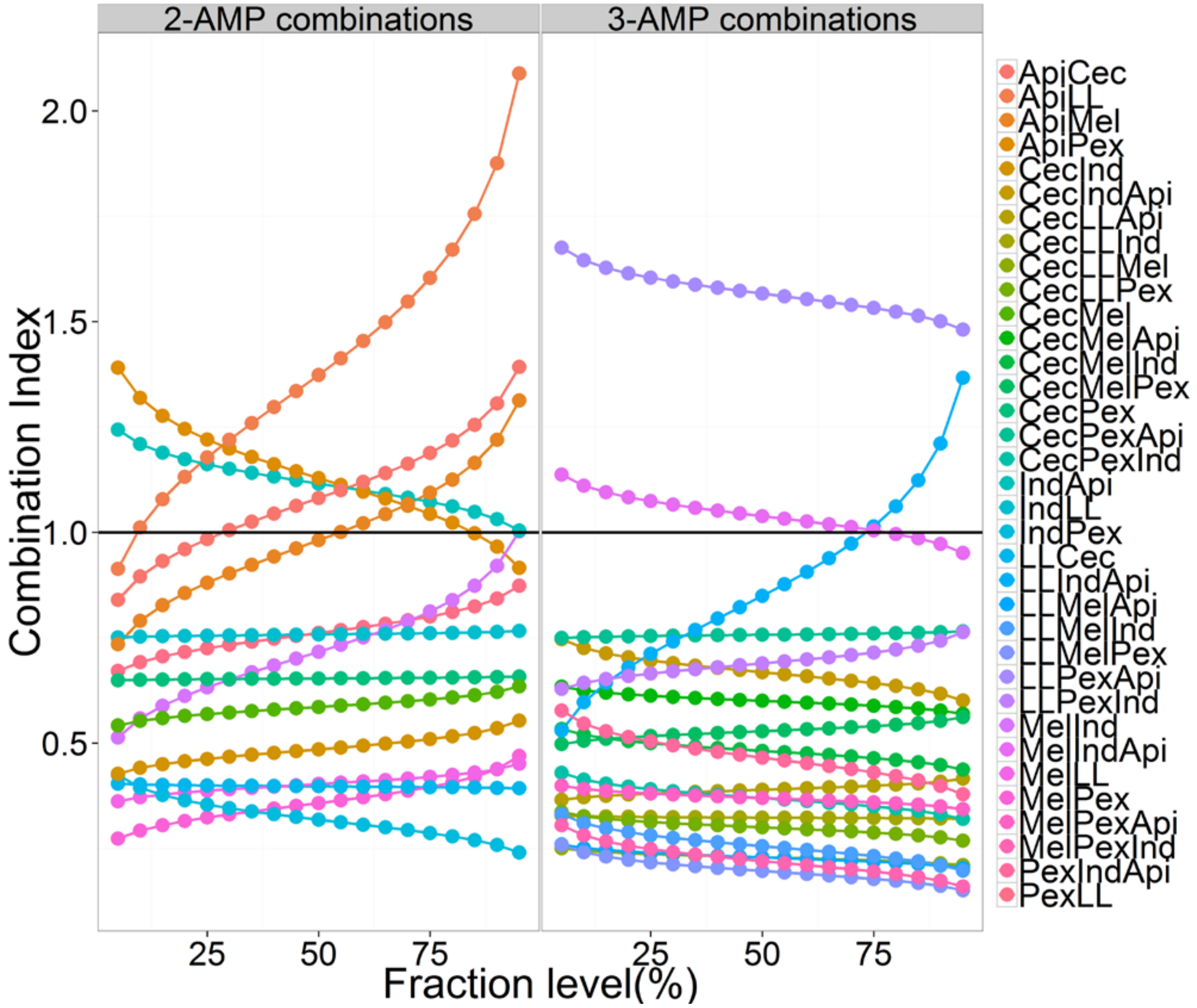

Table S1.

The details of AMPs used in current study.

Name	Abbreviations	Sequence	Molecular Weight	Source	MIC (µg/ml)
Cecropin A	Cec	KWKLFKKIEKVGQNIRDGIIKAGPAVAVVGQATQ IAK-NH_2	4004.8	AnaSpec	1
LL 17-29	LL	FKRIVQRIKDFLR	1719.1	AnaSpec	16
Melttin	Mel	GIGAVLKVLTTGLPALISWIKRKRQQ-NH_2	2846.5	AnaSpec	4
Pexiganan	Pex	GIGKFLKKAKKFGKAFVKILKK-NH_2	2477.2	Michael Zasloff	2
Indolicidin	Ind	ILPWKWPWWPWRR-NH_2	1906.3	AnaSpec	4
Apidaecin IB	Api	GNNRPVYIPQPRPPHPRL	2108.4	AnaSpec	4

Table S2. The four parameters zMIC, κ, ψ_{max} and ψ_{min} which are extracted from the fitting to the Hill function. Numbers in brackets represent lower and upper of 95% confidence intervals.

AMP(s)	zMIC[a] (lower,upper)	κ (lower,upper)	ψ_{max} (lower,upper)	ψ_{min} (lower,upper)
Api	46.40(39.1,52.94)	4.82(3.00,7.90)	0.18(0.13,0.23)	-3.29(-5.04,-1.91)
ApiCec	0.59(0.49,0.70)	3.97(3.29,4.80)	0.25(0.10,0.38)	-5.86(-5.97,-5.74)
ApiLL	13.73(11.63,16)	2.42(2.06,2.82)	0.25(0.18,0.31)	-7.29(-8.05,-6.55)
ApiMel	8.94(7.19,10.59)	2.98(2.47,3.53)	0.20(0.11,0.29)	-5.90(-6.23,-5.60)
ApiPex	11.00(10.58,11.44)	7.37(6.39,8.46)	0.32(0.26,0.36)	-5.82(-5.92,-5.73)
Cec	0.32(0.28,0.37)	6.02(5.17,6.84)	0.12(0.03,0.22)	-5.83(-5.89,-5.77)
CecInd	0.27(0.24,0.31)	4.65(4.19,5.15)	0.19(0.09,0.30)	-5.83(-5.90,-5.77)
CecLL	0.25(0.20,0.30)	6.09(5.07,7.27)	0.13(0.01,0.24)	-5.83(-5.91,-5.75)
CecMel	0.37(0.27,0.46)	5.12(3.69,7.06)	0.23(0.03,0.38)	-5.84(-5.95,-5.73)
CecPex	0.39(0.17,0.50)	5.84(4.06,7.79)	0.13(0.00,0.25)	-5.84(-5.94,-5.75)
Ind	5.20(4.14,6.28)	3.73(2.85,4.79)	0.30(0.17,0.42)	-5.78(-6.01,-5.56)
IndApi	11.44(9.56,13.34)	4.45(3.47,5.60)	0.28(0.18,0.37)	-5.79(-6.00,-5.58)
IndLL	5.11(4.68,5.56)	3.58(3.24,3.94)	0.27(0.22,0.32)	-5.93(-6.03,-5.84)
IndPex	1.94(1.67,2.12)	7.39(5.39,8.93)	0.24(0.18,0.30)	-5.83(-5.89,-5.77)
LL	10.23(8.70,11.82)	3.42(2.84,4.05)	0.23(0.14,0.31)	-6.08(-6.32,-5.86)
Mel	7.22(6.57,7.85)	4.13(3.79,4.51)	0.17(0.12,0.22)	-5.64(-5.74,-5.52)
MelInd	3.32(2.78,3.86)	2.69(2.30,3.05)	0.32(0.22,0.43)	-5.90(-6.08,-5.71)
MelLL	3.46(3.04,3.92)	3.34(2.91,3.81)	0.31(0.21,0.40)	-5.81(-5.96,-5.65)
MelPex	1.34(1.09,1.73)	3.23(2.67,3.75)	0.20(0.09,0.35)	-5.85(-6.01,-5.69)
Pex	4.19(3.86,4.54)	4.84(4.23,5.51)	0.27(0.20,0.35)	-5.83(-5.94,-5.71)
PexLL	4.03(3.38,4.68)	3.69(3.11,4.36)	0.27(0.14,0.38)	-5.83(-6.02,-5.64)
CecIndApi	0.74(0.70,0.78)	7.42(6.74,8.09)	0.21(0.15,0.26)	-5.82(-5.87,-5.78)
CecLLApi	0.35(0.32,0.38)	5.24(4.55,5.79)	0.17(0.10,0.23)	-5.82(-5.87,-5.78)
CecLLInd	0.25(0.06,0.28)	5.88(5.55,6.40)	0.06(0.00,0.11)	-5.83(-5.87,-5.79)
CecLLMel	0.14(0.07,0.30)	7.05(6.44,7.71)	0.00(0.00,0.10)	-5.83(-5.87,-5.78)
CecLLPex	0.28(0.12,0.31)	7.50(6.75,8.27)	0.07(0.00,0.12)	-5.83(-5.87,-5.79)
CecMelApi	0.65(0.61,0.68)	6.63(6.06,7.20)	0.22(0.16,0.28)	-5.83(-5.87,-5.78)
CecMelInd	0.51(0.45,0.57)	7.15(5.19,8.60)	0.20(0.14,0.27)	-5.84(-5.89,-5.79)
CecMelPex	0.49(0.46,0.52)	5.21(4.65,5.79)	0.30(0.24,0.36)	-5.83(-5.87,-5.78)
CecPexApi	0.69(0.63,0.77)	5.79(5.06,6.67)	0.16(0.10,0.23)	-5.82(-5.87,-5.76)
CecPexInd	0.27(0.25,0.29)	7.97(6.80,8.89)	0.32(0.20,0.43)	-5.97(-6.07,-5.88)
LLIndApi	5.99(5.59,6.44)	2.32(2.16,2.49)	0.42(0.38,0.47)	-5.91(-6.05,-5.77)
LLMelApi	3.18(2.94,3.50)	4.67(4.08,5.42)	0.24(0.19,0.29)	-5.80(-5.87,-5.73)
LLMelInd	2.00(1.83,2.17)	5.65(5.22,6.03)	0.09(0.06,0.14)	-5.79(-5.85,-5.74)
LLMelPex	1.58(1.42,1.80)	7.19(6.13,8.99)	0.15(0.10,0.20)	-5.77(-5.82,-5.72)
LLPexApi	14.94(13.89,16.15)	4.90(4.22,5.68)	0.34(0.30,0.38)	-5.89(-5.99,-5.78)
LLPexInd	3.61(3.35,3.87)	3.65(3.36,3.96)	0.29(0.23,0.34)	-5.86(-5.95,-5.77)

MelIndApi	8.62(7.41,9.98)	4.47(3.89,5.15)	0.15(0.06,0.22)	-5.87(-6.03,-5.70)
MelPexApi	2.71(2.18,3.18)	5.21(4.02,6.60)	0.13(0.05,0.21)	-5.78(-5.89,-5.68)
MelPexInd	1.58(1.46,1.69)	8.05(7.01,8.92)	0.19(0.14,0.24)	-5.83(-5.87,-5.78)
PexIndApi	3.82(3.42,4.15)	6.30(4.97,7.39)	0.28(0.24,0.32)	-5.83(-5.89,-5.77)

a, The unit of zMIC is not μg/ml in this table, but the times of original MIC of each AMP(s). We set the initial concentration of AMP(s) to 100 times of original MIC when we were doing the experiment.
